# Exploring the Effects of Oral Calcium Bolus Supplementation on Serum Minerals and Energy Balance Indicators in Simmental Cows Fed a Prepartum Anionic Diet

**DOI:** 10.3390/vetsci12111032

**Published:** 2025-10-24

**Authors:** Salih Çelik, Habip Muruz, Sude Bayram, Zehra Selçuk, Mehmet Akif Yörük

**Affiliations:** 1 Tokat Provincial Directorate of Agriculture and Forestry, 60100 Tokat, Türkiye; celik.salih@tarimorman.gov.tr; 2 Department of Animal Nutrition and Nutritional Diseases, Faculty of Veterinary Medicine, Ondokuz Mayıs University, 55200 Samsun, Türkiye; zselcuk@omu.edu.tr (Z.S.); mayoruk@omu.edu.tr (M.A.Y.); 3 Department of Animal Nutrition and Nutritional Diseases, Graduate Education Institute, Ondokuz Mayıs University, 55200 Samsun, Türkiye; 22281559@stu.omu.edu.tr

**Keywords:** dairy cows, prepartum DCAD, calcium boluses, hypocalcemia

## Abstract

**Simple Summary:**

Calcium deficiency around calving is one of the most common problems in dairy cows, and farmers often use oral calcium boluses to prevent it. In this study, we tested a calcium bolus that contained three different calcium sources and vitamin D in Simmental cows, which are a dual-purpose breed. All cows were fed a special diet before calving to help them use calcium more efficiently, and at calving, their blood calcium levels were already normal. Half of the cows received two calcium boluses, one at calving and another 24 h later, while the other half did not receive any boluses. Blood samples were taken during the first two days and again up to two weeks after calving to measure calcium, phosphorus, magnesium, glucose, and ketone bodies. The results showed that the calcium boluses caused only a small and short-lived rise in blood calcium, and there were no clear differences between treated and untreated cows for any of the measured blood values. This study suggests that, in well-managed Simmental herds already receiving proper pre-calving diets, extra calcium supplementation may not be necessary when cows have normal calcium levels at calving. These findings can help farmers use supplements more effectively and avoid unnecessary costs.

**Abstract:**

Calcium disorders remain a major challenge during the transition period of dairy cows, and oral calcium supplements are widely used to prevent postpartum hypocalcemia. This study evaluated the effects of administering an oral Ca-bolus containing calcium sulfate, acetate, and formate on postpartum mineral status and energy balance in multiparous Simmental cows. Twenty cows (mean parity 3.5 ± 0.51) were fed a prepartum diet with a negative dietary cation–anion difference (DCAD) and enrolled at calving if body condition score was between 3.0 and 3.5 and urine pH ranged from 6.2 to 6.8 during the wk before parturition. Animals were blocked by parity and randomly assigned to a control group (*n* = 10) or to a treatment group (*n* = 10) receiving two oral boluses (each 175 g; 45.14 g calcium plus 40,250 IU vitamin D_3_), administered immediately after calving and 24 h later. Blood samples were collected up to 48 h postpartum for calcium, phosphorus, and magnesium analyses, and up to 14 d postpartum for glucose and β-hydroxybutyrate. Both groups were normocalcemic at calving, and no significant treatment effects were detected for any parameter at any time point. However, a short-lived numerical increase in calcium was observed 6 h after bolus administration. These results suggest that additional oral Ca supplementation may not be required in well-managed Simmental cows receiving a prepartum negative DCAD diet.

## 1. Introduction

During the transition period, particularly multiparous dairy cows experience a marked decline in calcium (Ca) homeostasis due to rapid Ca loss around calving, thereby increasing the risk of milk fever as blood Ca concentrations drop below physiological levels [1,2]. Depending on the severity of this decline, hypocalcemia can manifest either as subclinical or clinical, the latter occurring when blood Ca becomes insufficient to support nerve and muscle function [3]. Clinical hypocalcemia is of low incidence (1–5%) compared to subclinical hypocalcemia (41–54%) in multiparous cows (SCH; serum Ca ≤ 8.0 mg/dL) [4]. Subclinical hypocalcemia is associated with impaired neutrophil function, an increased risk of diseases such as subclinical ketosis, displaced abomasum, metritis, and subclinical endometritis, reduced dry matter intake (DMI) and milk yield, and decreased odds of pregnancy at first AI [5,6,7]. Evidence also indicates that SCH can affect the metabolic status of cows by reducing insulin concentrations and increasing fatty acid mobilization, hepatic lipid accumulation, and blood glucose concentrations [4,8,9,10,11].

Feeding anionic salts to close-up cows is a widely implemented strategy in dairy herds and has been shown to effectively reduce the incidence of clinical hypocalcemia [12]. This approach induces a state of mild compensated metabolic acidosis, thereby enhancing parathyroid hormone (PTH) responsiveness and improving Ca mobilization at calving [13]. Nevertheless, SCH remains prevalent even in cows fed anionic salts during the close-up period [14,15]. To further mitigate the adverse effects of SCH, peripartum oral Ca-bolus supplementation has been proposed as an additional management strategy. Administering Ca-containing boluses immediately after calving, followed by repeated doses at 12 to 24 h intervals, represents a practical and effective method to increase serum Ca concentrations during the early postpartum period [16,17]. However, the efficacy of this approach depends on bolus composition, dissolution properties, and frequency of administration [18,19]. Commercial and non-commercial Ca-bolus formulations vary widely in their composition, and several combinations have been evaluated, mostly in Holstein cows. Reported formulations include a combination of Ca-chloride and sulfate [17,20,21], Ca-chloride, sulfate, propionate, and lactate [22], Ca-chloride, propionate, and fumarate [15], and Ca-propionate, carbonate, and formate [23]. Ca-chloride dissociates rapidly, providing a prompt but transient rise in blood Ca [24], whereas Ca-sulfate, Ca-acetate, and Ca-formate dissolve more slowly, potentially sustaining absorption over longer periods [25,26,27]. Some formulations also include vitamin D3 to stimulate active intestinal Ca transport.

Despite the widespread use of oral Ca-boluses, critical gaps remain in understanding the comparative efficacy of different Ca salt formulations, particularly in cows already managed with prepartum negative DCAD diets. Additionally, most research assessing prophylactic Ca supplementation has focused on high-yielding Holstein cows [12,15], whereas information regarding dual-purpose breeds such as Simmental remains limited. Simmental cows exhibit distinct metabolic adaptations during the transition period compared with Holsteins [28,29], and previous studies have shown that their blood Ca concentrations can approach threshold levels around calving [30,31]. However, to our knowledge, no controlled trial has specifically investigated whether Ca-bolus formulations provide additional benefits to Ca homeostasis in Simmental cows fed acidogenic prepartum diets. Therefore, we hypothesized that oral supplementation with a Ca-bolus containing Ca-sulfate, acetate, and formate, in conjunction with an acidogenic prepartum diet, would enhance postpartum Ca homeostasis in Simmental cows. Specifically, the objective of this study was to assess the impact of oral Ca-bolus supplementation on Ca homeostasis during the first 48 h after calving and its associated effects on mineral and metabolic balance in Simmental cows. By addressing these questions in Simmental cows—an underrepresented dual-purpose breed—this study aims to provide novel insights into breed-specific responses and to offer practical guidance for tailoring supplementation strategies beyond the Holstein-centric evidence base.

## 2. Materials and Methods

This study was conducted between April and October 2024 at the YUSİF Dairy Farm located in the Kurugökçe District of Atakum, Samsun, Türkiye. The farm maintained a mean herd size of approximately 200 lactating Simmental cows—the predominant breed on the farm—with an average standardized 305-day milk yield of 6500 kg per cow. The herd was selected to represent a well-managed commercial system characterized by uniform feeding, housing, and management practices, thereby ensuring experimental consistency and minimizing environmental variation between treatment groups. All animal procedures were performed with the approval of the Ondokuz Mayıs University Animal Experiments Ethics Committee (Protocol No: 2023/98).

### 2.1. Animals and Experimental Design

Twenty multiparous Simmental cows (three to four lactations) were included in the study. The inclusion criteria were a body condition score (BCS; 1–5 scale; [32]) between 3.0 and 3.5 at calving, and a urine pH between 6.2 and 6.8 [16], measured 7 to 10 d before the expected calving date. Cows with twins or dystocia, or presenting clinical signs of disease (e.g., milk fever, retained placenta, displaced abomasum, metritis, or clinical mastitis), were not included in the study or were to be excluded if diagnosed after enrollment; however, no such cases occurred during the experimental period. Eligible cows were blocked by parity (third vs. fourth lactation) prior to random assignment to treatment groups to control for potential variability associated with lactation number. and randomly assigned to 1 of 2 groups within a randomized complete block design. Randomization was conducted using a computer-generated random number list in Microsoft Excel (Microsoft Corp., Redmond, WA, USA). The treatments were as follows: one oral Ca-bolus (45 g of Ca per bolus) immediately after calving (d 0), and a second Ca-bolus 24 h (±30 min) later (Ca-bolus group; *n* = 10), or no Ca-bolus (control group; *n* = 10). Farm employees were blinded to the cow supplementation-group assignment. The Ca supplement used in the treatment group was a commercial oral bolus (Calstorm^®^, BAYFEED Hayvancılık Gıda Yem San. ve Tic. Ltd. Şti., Gaziemir–İzmir, Türkiye), providing approximately 45 g of elemental Ca per cow administration (Table 1). The total Ca content of the bolus was within the range considered adequate to achieve ruminal Ca concentrations sufficient for effective absorption across the rumen epithelium, as previously described by Schröder et al. [33]. Boluses were administered using a standard applicator, and cows were observed for 5–10 min post-dosing to ensure that the bolus was not regurgitated.

### 2.2. Housing and Herd Management

All experimental cows were housed and managed under the same environmental and nutritional conditions. Cows were housed indoors throughout the experimental period. During the dry period, cows were kept in a free-stall system. Animals were monitored every 30 min by trained farm staff for signs of imminent parturition. At the first signs of parturition, cows were moved to individual straw-bedded maternity pens (40 m^2^ each). Calves were separated from their dams immediately after birth. Postpartum cows remained in the calving pen for 48 h following parturition before being transferred to free-stall lactation pens. Farm personnel monitored cows in the calving and lactation pens daily during the morning lock-up for signs of health disorders. The barn was naturally ventilated through roof and wall slats, and cows had free access to clean drinking water via the standard drinking system in the calving pen, which was also available during treatment administration. During lactation, cows were milked twice daily at 0600 and 1700 h in a 30-stall milking parlor.

### 2.3. Diets and Feeding

All cows received identical prepartum and postpartum diets, provided twice daily as total mixed rations (TMR). Prepartum and postpartum diets (Table 2) were formulated according to NRC [34] nutrient requirements. The prepartum diet was formulated for dry cows with an estimated body weight (BW) of 680 kg and a predicted dry matter intake (DMI) of 11.6 kg/d, and was designed to achieve a negative DCAD of −109 mEq/kg dry matter (DM). It was offered from 21 d before the expected calving date. The postpartum diet was formulated for cows at 14 days in milk (DIM), assuming a BW of 620 kg and a daily milk yield of 25 kg, with target milk components of 3.7% fat and 3.0% protein, and a predicted DMI of 19.2 kg/d. Feed was pushed up two to three times daily to stimulate intake.

### 2.4. Measurements, Sampling and Calculations

Representative samples of each diet type were collected directly from the feed bunk and subjected to chemical analysis. The dry matter content of the rations was determined by drying samples in a forced-air oven at 105 °C for 4 h. Ash content was measured by incineration at 550 °C in a muffle furnace for 4 h. Nitrogen content was determined using the Kjeldahl method, and crude protein was calculated as N × 6.25. Ether extract (crude fat) was analyzed according to AOAC [35] procedures. Cell wall fractions, including neutral detergent fiber (NDF) and acid detergent fiber (ADF), were determined using an ANKOM 200 Fiber Analyzer (ANKOM Technology Corp., Fairport, NY, USA), following the methodology of Van Soest et al. [36]. Net energy for lactation (NEL), mineral contents (Ca, P, Mg), and DCAD were calculated based on tabular values provided by NRC [34]. The DCAD was calculated according to Tucker et al. [37] using the following equation:

DCAD (mEq/kg DM) = [(Na^+^ + K^+^) − (Cl^−^ + S2^−^)].

Approximately 10 mL of blood was collected from the coccygeal vein using vacuum tubes with clot activator and 21 G needles (25 × 0.8 mm) at seven predefined time points for mineral analysis: immediately after calving (0 h) and at 6, 12, 24, 30, 36, and 48 h postpartum. For serum glucose analysis, samples were collected on d 0, 1, 2, 7, and 14 postpartum. All samples were allowed to clot at room temperature for 20 min and centrifuged at 2000× *g* for 15 min (LC-04B centrifuge, Zenith Lab, Jintan, Jiangsu, China). Serum was separated and stored at −20 °C until laboratory analysis, which was performed within 10 mo of collection. Serum Ca, P, Mg, and glucose concentrations were determined using a multi-parameter clinical chemistry analyzer (Fujifilm Dri-Chem NX500, Fujifilm Corp., Tokyo, Japan) with commercial reagent kits. Blood concentrations of β-hydroxybutyrate (βHBA) were measured on d 0, 1, 2, 7, and 14 of lactation using a portable analyzer (Vet TD-4235 β-Ketone Monitoring System, Taiwan) with specific reagent strips, from a drop of blood obtained by pricking the tip of the tail with a 25 × 0.8 mm needle. In the present study, subclinical hypocalcemia was defined as serum Ca ≤ 8.0 mg/dL [4], subclinical ketosis as βHBA ≥ 1.2 mmol/L [38,39], hypophosphatemia as serum P ≤ 4.0 mg/dL [40], and hypomagnesemia as serum Mg ≤ 2.0 mg/dL [41].

### 2.5. Statistical Analysis

All statistical analyses were conducted using SPSS Statistics software (Version 25.0; IBM Corp., Armonk, NY, USA). Blood metabolites data were analyzed using a two-way repeated measures ANOVA. The statistical model included the fixed effects of treatment (oral Ca-bolus: supplemented vs. control), time (repeated measures at 0, 6, 12, 24, 30, 36, and 48 h postpartum for serum Ca, P, and Mg; and at d 0, 1, 2, 7, and 14 for glucose and βHBA, and their interaction (treatment × time). Prior to analysis, data were checked for normal distribution using the Shapiro–Wilk test and for homogeneity of variances using the Levene test. The Mauchly test was applied to verify the assumption of sphericity; when this assumption was violated, the Greenhouse–Geisser correction was used to adjust the degrees of freedom. When significant main effects were detected, post hoc pairwise comparisons were performed using Bonferroni-adjusted estimated marginal means to control the Type I error rate. All results are presented as mean ± standard error of the mean (SEM), and statistical significance was declared at *p* < 0.05.

## 3. Results

### 3.1. Serum Mineral Profiles

**Figure 1:**
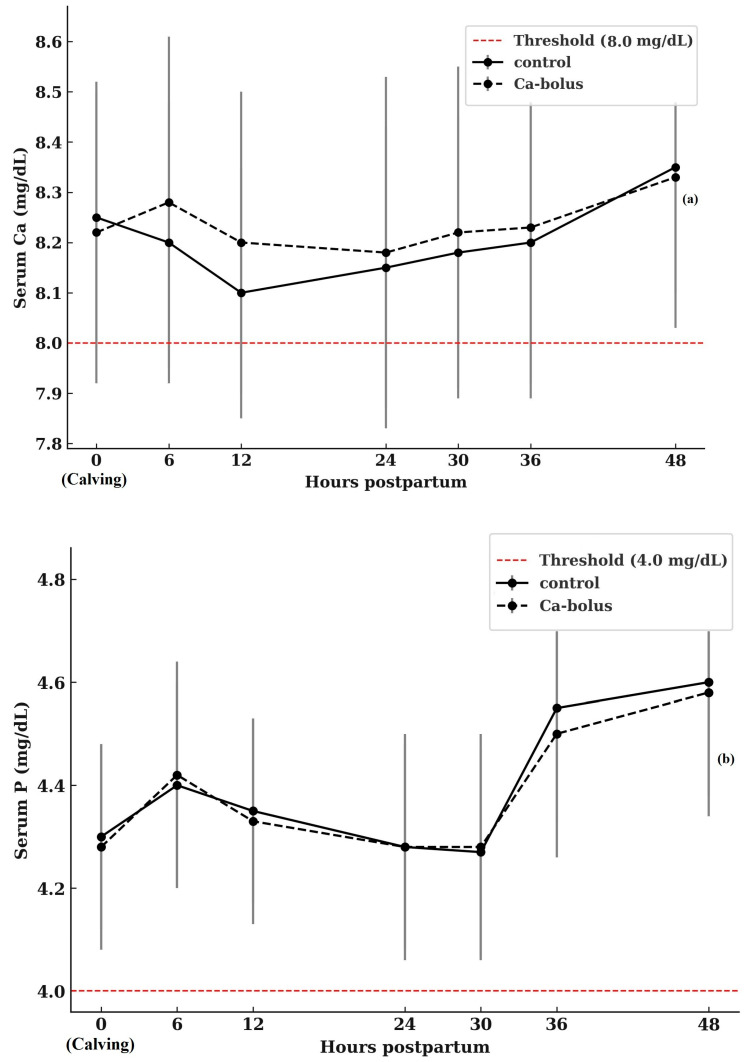

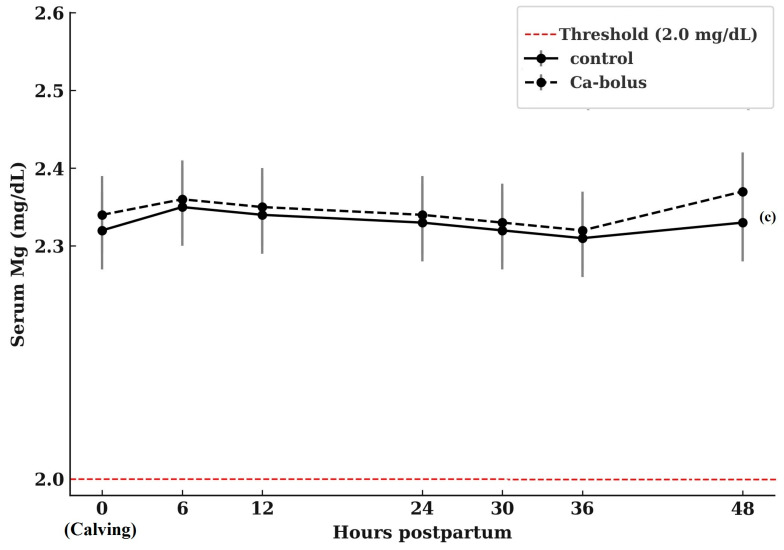
Serum Ca (**a**), P (**b**), and Mg (**c**) concentrations in multiparous Simmental cows in the control (*n* = 10) and oral Ca-bolus (*n* = 10) groups over time postpartum (0 to 48 h). The red dashed line represents the threshold for subclinical hypocalcemia (≤8.0 mg/dL; [4]), hypophosphatemia (≤4.0 mg/dL; [40]), and hypomagnesemia (≤2.0 mg/dL, [41]). Error bars: ± standard deviation. There was no significant group × time interaction (*p* > 0.05) or main effect of time (*p* > 0.05). Error bars represent ± standard deviation and indicate the degree of inter-individual variation at each time point.

Patterns of serum Ca concentrations over the study period are presented in Figure 1a. At calving (0 h), baseline Ca concentrations did not differ between treatment groups, with control cows averaging 8.25 ± 0.75 mg/dL and Ca-bolus-treated cows averaging 8.20 ± 0.48 mg/dL (*p* > 0.05). Although statistical differences were not observed, a transient numerical increase in serum Ca concentration was evident at 6 h postpartum in the Ca-bolus group compared with the control (8.29 ± 0.82 vs. 8.18 ± 0.50 mg/dL, *p* > 0.05). Blood Ca concentrations did not differ between groups at any subsequent sampling time, and neither a main effect of time nor a group × time interaction was detected. An increase in serum P levels was observed in all cows during the first two days postpartum (Figure 1b). However, the treatment effect was not statistically significant, and no treatment × time interaction was detected throughout the 2 DIM period. The treatments did not affect Mg concentrations within the first 48 h postpartum (Figure 1c). A slight increase in Mg levels was observed in all cows on d 2 compared to d 1 (*p* > 0.05).

### 3.2. Energy-Related Blood Metabolites

**Figure 2:**
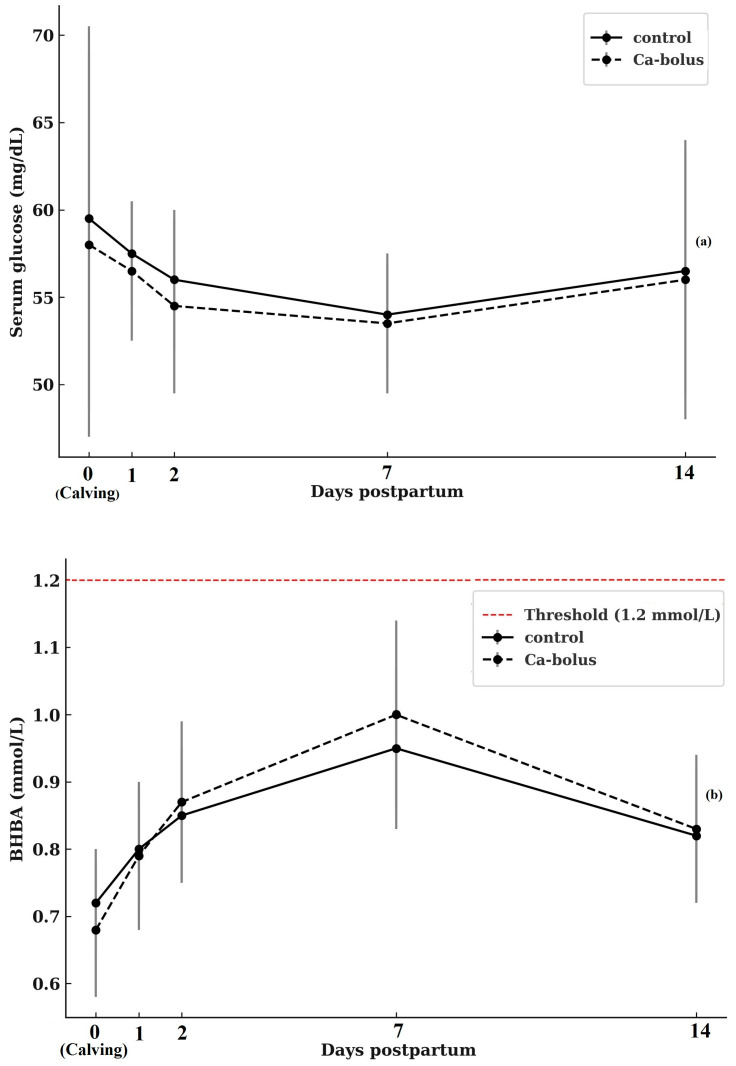
Serum glucose (**a**) and ßHBA concentration (**b**) over time (d 0, 1, 2, 7, and 14 postpartum) for the control (*n* = 10) and oral Ca-bolus (*n* = 10) groups. The red dashed line represents subclinical ketosis (≥1.2 mmol/L, McArt et al. [38]; Shin et al. [39]. Error bars: ± standard deviation. There was no significant group × time interaction (*p* > 0.05) or main effect of time (*p* > 0.05) on serum glucose concentrations. There was significant main effect of time on βHBA (*p* < 0.05) Error bars represent ± standard deviation and indicate the degree of inter-individual variation at each time point.

Serum glucose concentrations during the first 14 d postpartum are shown in Figure 2a. At calving (d 0), mean glucose values did not differ between groups, averaging 59.4 ± 20.83 mg/dL in control cows and 58.0 ± 10.55 mg/dL in Ca-bolus cows. No treatment effects were detected at any time point (*p* > 0.05). Both groups exhibited a decline in glucose concentrations during the first 2 d postpartum, reaching nadir values of 55.9 ± 7.56 mg/dL (control) and 54.0 ± 10.50 mg/dL (Ca-bolus). Thereafter, glucose concentrations stabilized around 53–55 mg/dL until d 7, followed by a modest rebound toward baseline levels by d 14 (56.4 ± 12.92 vs. 56.3 ± 8.20 mg/dL for control and Ca-bolus, respectively; *p* > 0.05). Neither a main effect of time nor a treatment × time interaction was observed (*p* > 0.05). Collectively, these results indicate that oral Ca-bolus supplementation did not alter glucose dynamics in early lactation, as both groups displayed a similar temporal pattern characterized by an initial postpartum decline and partial recovery by two weeks after calving.

Serum βHBA concentrations during the first 14 d postpartum are shown in Figure 2b. At calving (d 0), baseline βHBA concentrations were comparable between groups (0.71 ± 0.14 mmol/L in controls and 0.67 ± 0.21 mmol/L in Ca-bolus cows; *p* > 0.05). No treatment effects or treatment × time interactions were observed. However, a significant main effect of time was detected (*p* < 0.05). Both groups exhibited a progressive rise in βHBA from calving to d 7, reaching peak values of 0.90 ± 0.25 mmol/L (control) and 0.94 ± 0.23 mmol/L (Ca-bolus), followed by a slight decline by d 14 (0.77 ± 0.15 mmol/L and 0.80 ± 0.20 mmol/L, respectively). Importantly, mean βHBA concentrations in both groups remained below the threshold for subclinical ketosis (1.2 mmol/L) throughout the study, indicating that neither group experienced a clinically relevant negative energy balance during early lactation.

## 4. Discussion

The transition period, encompassing the final weeks of gestation and the first weeks of lactation, is characterized by profound metabolic adjustments that challenge the capacity of dairy cows to maintain mineral and energy homeostasis. Among the most critical adaptations is the regulation of Ca metabolism, which interacts closely with energy balance to support the onset of lactation. In this study, the combined effects of prepartum negative DCAD feeding and postpartum oral Ca-bolus supplementation were evaluated in Simmental cows. The findings demonstrated that while temporal changes in Ca, glucose, and βHBA concentrations were consistent with the expected metabolic shifts in early lactation, oral Ca-bolus supplementation did not significantly influence these dynamics compared with control cows.

One of the strengths of this study was the oral administration of Ca-boluses to cows immediately after calving, allowing each cow to serve as the experimental unit. This study design allowed cows from both treatment and control groups to be mixed in the same batch and managed identically, eliminating the influence of barn and/or management practices on the study variables. However, this study presents several limitations that should be acknowledged when interpreting the results. One of the weaknesses of this study was the lack of measurement of individual DMI. The study was conducted on a commercial dairy farm; therefore, it was not possible to assess DMI. In addition, the small sample size (*n* = 10 per group) limits statistical power to detect subtle effects, and conducting the study on a single farm may restrict generalizability. Breed-specific physiology and Ca source solubility should be considered when extrapolating results.

### 4.1. Calcium, Phosphorus, and Magnesium

The present findings indicate that oral Ca-bolus supplementation did not provide a sustained benefit in circulating Ca concentrations compared with controls. Although a transient numerical increase in serum Ca was observed at 6 h in the supplemented group, this effect diminished rapidly. This pattern aligns with previous studies reporting that oral Ca boluses typically induce only a short-lived rise in serum Ca, primarily when formulations are dominated by highly soluble salts such as Ca-chloride [15,21,42]. In contrast, the absence of a pronounced peak in our study likely reflects the use of a bolus containing Ca-formate, Ca-acetate, and Ca-sulfate. These salts have slower dissolution and absorption profiles compared with Ca-chloride [27], which may help moderate fluctuations in circulating Ca. The lack of a clear treatment effect in the present study may reflect both the influence of an acidogenic prepartum diet and breed-specific physiology. The key factor is the prepartum negative DCAD diet. Numerous studies confirm that negative DCAD feeding reduces the risk of clinical hypocalcemia and stabilizes Ca homeostasis postpartum [9,22]. In the present study, the stable serum Ca levels across both groups suggest that the negative DCAD diet provided substantial protection against hypocalcemia, thereby limiting the potential added value of oral bolus supplementation. Nevertheless, it should be noted that breed-specific physiology may influence these dynamics. Much of the existing literature has focused on Holstein cows, which are known to exhibit higher incidences of clinical and SCH due to their elevated milk yield and Ca secretion [8]. Simmental cattle tend to have a moderate milk yield and a lower incidence of metabolic disorders compared with specialized dairy breeds [43]. These characteristics likely resulted in a lower risk of postpartum Ca imbalance and may have reduced the physiological response to Ca supplementation. Another possible explanation is that the cows used in the present study were in their third and fourth lactations, and therefore, their Ca homeostatic regulatory mechanisms were more active. This may have limited the effect of Ca supplementation. These mechanisms—particularly calcitonin secretion and renal Ca excretion—can rapidly eliminate excess Ca supplied from external sources [44]. On the other hand, despite including vitamin D_3_ in our bolus formulation, no sustained response was observed, aligning with the findings of Shock et al. [45], which suggest that vitamin D_3_ has limited short-term effectiveness in improving postpartum Ca dynamics. Overall, the present findings indicate that cows were normocalcemic at the onset of lactation and that Ca-bolus supplementation had only a limited physiological effect. These results suggest that routine Ca-bolus supplementation may have limited benefit in well-managed herds with adequate prepartum Ca balance and acidogenic diets. However, further studies are warranted to define the physiological thresholds at which oral Ca supplementation provides measurable benefits across breeds and management systems.

Blood concentrations of P and Mg during the early postpartum period remained within the physiological ranges previously reported [46,47,48]. Moreover, no significant differences between groups were detected over time, consistent with earlier findings by these investigators. The stability of serum P can be explained by the physiological regulation of P metabolism under normocalcemic conditions. As described by Goff [44], reduced PTH secretion in the absence of hypocalcemia limits urinary and salivary P losses while maintaining intestinal absorption and homeostatic balance. This regulatory mechanism likely accounts for maintaining serum P concentrations within the normal range in both groups throughout the observation period. Supplementation with high quantities of Ca has the potential to interfere with Mg metabolism by reducing gastrointestinal absorption and increasing renal Mg excretion [4]. In this context, Martinez et al. [49] reported a decline in blood Mg concentrations following administration of Ca-chloride. According to the authors, the high solubility of Ca-chloride induces a sharp and transient hypercalcemia, which may stimulate urinary Mg excretion and thereby disrupt Mg balance. In the present study, however, serum Mg concentrations remained within the physiological range. This discrepancy is likely attributable to the lower Ca dose per bolus (45 g) and the inclusion of less soluble Ca salts in our formulation, which did not elevate ruminal Ca to a level sufficient to impair Mg absorption. These findings suggest that the impact of oral Ca supplementation on Mg homeostasis depends not only on the quantity of Ca administered but also on the solubility characteristics of the Ca source used.

### 4.2. Energy Metabolism Biomarkers

In the present study, serum glucose concentrations followed a typical postpartum pattern, characterized by a decline during the first 2 d after calving, stabilization through the first week, and a modest rebound toward baseline by d 14. This temporal trend is consistent with the expected metabolic adaptations to the onset of lactation, when glucose demand for lactose synthesis increases sharply [50]. Importantly, oral Ca-bolus supplementation did not influence glucose concentrations. Previous studies have demonstrated that SCH may impair pancreatic insulin secretion by reducing Ca^2+^ influx, thereby limiting glucose uptake in peripheral tissues and increasing circulating glucose levels [7,51,52]. However, all cows in the present study maintained normocalcemia, which likely explains why blood glucose concentrations followed expected temporal patterns without significant differences between groups. Moreover, although hypocalcemia has been associated with reduced gastrointestinal motility and impaired feed intake [16,53], maintaining serum Ca near normal thresholds in both groups likely prevented secondary effects on nutrient utilization and overall energy metabolism. Collectively, these findings support the view that the relationship between Ca status and glucose homeostasis is largely indirect, mediated through feed intake and rumen function rather than direct endocrine pathways. Beyond these mechanisms, breed-specific physiology is an important determinant of the relationship between Ca status and energy balance. Studies in Holstein cows have shown that hypocalcemia is associated with more severe negative energy balance (NEB), increased non-esterified fatty acid mobilization, and altered glucose partitioning [54,55]. In contrast, it is known that energy deficit is less in dual-purpose breeds, such as Simmental, thereby reducing the need for metabolic adaptations that markedly depress glucose [56]. Thus, breed differences in metabolic load likely contributed to the absence of a detectable treatment effect on glucose dynamics in the present study.

Similarly, βHBA concentrations increased progressively until d 7, followed by a slight decline toward d 14. This trajectory reflects the transient NEB commonly observed in early lactation [57]. Despite the rise, mean βHBA values remained well below the subclinical ketosis threshold of 1.2 mmol/L [38,39], which is primarily derived from studies in Holstein and Jersey cows. Given the physiological and productive differences in dual-purpose breeds such as Simmental, future studies should aim to establish breed-specific thresholds to improve the interpretation of metabolic health indicators. Nevertheless, the significant main effect of time highlights the metabolic challenge imposed by the transition period, which may be further exacerbated under higher-yielding conditions or suboptimal nutritional management. Although hypocalcemia is known to exacerbate NEB by reducing feed intake and increasing fat mobilization [16], the absence of profound hypocalcemia in this study likely minimized any indirect effects of Ca supplementation on βHBA. This finding is consistent with previous reports showing minimal or inconsistent effects of Ca-bolus supplementation on ketone body concentrations, particularly in herds managed with effective prepartum nutritional strategies [15,58]. Breed physiology also plays an important role, as Simmental cows mobilize body reserves more gradually [28,59] and consequently maintain a more favorable energy homeostasis [60], adapting more effectively to postpartum negative energy balance than Holstein or crossbred counterparts [61]. This metabolic resilience likely contributed to maintaining βHBA values below risk thresholds, regardless of Ca supplementation. In summary, the stable energy profile in this study likely reflects both breed physiology and the protective effect of DCAD feeding.

## 5. Conclusions

In conclusion, oral Ca-bolus supplementation did not significantly influence serum Ca, glucose, or ßHBA concentrations in Simmental cows fed a prepartum negative DCAD diet. While temporal changes in these parameters reflected the expected postpartum metabolic challenges, all values remained within physiological thresholds, and no evidence of clinically relevant hypocalcemia or ketosis was detected. These findings suggest that effective prepartum dietary management may be sufficient to support postpartum metabolic adaptation in dual-purpose breeds. However, results of the present study are based on a small sample of animals, and further studies are needed to confirm these findings.

## Figures and Tables

**Table 1:** Composition of the calcium bolus used in the study ^1^.

Component Name ^2^	Calcium Content (%)	Amount of Calcium Source (mg/kg)	Calcium Supplied (mg/kg Ca)	Per Bolus (175 g)
Calcium sulfate anhydrous	28.0	346,500	97,000	16.97 g
Calcium formate	29.0	431,000	125,000	21.87 g
Calcium acetate	23.7	152,000	36,000	6.30 g
Total calcium content	–	258,000	–	45.14 g

^1^ As declared by the manufacturer; ^2^ The bolus contains 40,250 IU of vitamin D_3_.

**Table 2:** Ingredients and nutrient composition of prepartum and postpartum diets.

Ingredient (% of DM)	Prepartum	Postpartum
Corn silage	28.5	21.5
Alfalfa silage	–	5.2
Alfalfa hay	–	16.9
Meadow hay	15.4	–
Wheat straw	7.9	–
Prefresh concentrate mix	45.6	–
Postfresh concentrate mix	–	46.5
Flaked corn	–	8.6
Vitamin–mineral premix ^1^	0.52	0.34
Calcium carbonate	0.35	0.29
Magnesium oxide	–	0.19
Mycotoxin binder	0.43	0.24
Salt	–	0.24
Ammonium chloride	1.3	–
Nutrient composition (DM basis)		
NE_L_ ^2^ MJ/kg DM	6.0	6.9
Crude protein, %	13.1	16.1
Crude ash, %	7.5	6.8
Ether extract, %	2.7	3.5
Neutral detergent fiber, %	33.3	30.6
Acid detergent fiber, %	19.6	17.9
Ca ^2^, %	0.31	0.94
P ^2^, %	0.22	0.35
Mg ^2^, %	0.14	0.29
Na ^2^, %	0.09	0.27
K ^2^, %	0.84	1.33
Cl ^2^, %	1.13	0.54
S ^2^, %	0.16	0.20
DCAD ^2^ (mEq/kg DM)	–109.09	243.48

^1^ Provided per kg of dry matter: 1,500,000 IU vitamin A, 450,000 IU vitamin D, 17,000 mg vitamin E, 3000 mg antioxidant, 10,000 mg Mn, 150 mg I, 16,000 mg Zn, 800 mg Fe, 4000 mg Cu, 120 mg Co, 80 mg Se. ^2^ Calculated based on tabular value of NRC [34].

## Data Availability

The original contributions presented in this study are included in the article. Further inquiries can be directed to the corresponding author.
